# DNA Sequences at a Glance

**DOI:** 10.1371/journal.pone.0079922

**Published:** 2013-11-21

**Authors:** Armando J. Pinho, Sara P. Garcia, Diogo Pratas, Paulo J. S. G. Ferreira

**Affiliations:** Signal Processing Lab, IEETA/DETI, University of Aveiro, Aveiro, Portugal; University of North Carolina at Charlotte, United States of America

## Abstract

Data summarization and triage is one of the current top challenges in visual analytics. The goal is to let users visually inspect large data sets and examine or request data with particular characteristics. The need for summarization and visual analytics is also felt when dealing with digital representations of DNA sequences. Genomic data sets are growing rapidly, making their analysis increasingly more difficult, and raising the need for new, scalable tools. For example, being able to look at very large DNA sequences while immediately identifying potentially interesting regions would provide the biologist with a flexible exploratory and analytical tool. In this paper we present a new concept, the “information profile”, which provides a quantitative measure of the local complexity of a DNA sequence, independently of the direction of processing. The computation of the information profiles is computationally tractable: we show that it can be done in time proportional to the length of the sequence. We also describe a tool to compute the information profiles of a given DNA sequence, and use the genome of the fission yeast *Schizosaccharomyces pombe* strain 972 h^−^ and five human chromosomes 22 for illustration. We show that information profiles are useful for detecting large-scale genomic regularities by visual inspection. Several discovery strategies are possible, including the standalone analysis of single sequences, the comparative analysis of sequences from individuals from the same species, and the comparative analysis of sequences from different organisms. The comparison scale can be varied, allowing the users to zoom-in on specific details, or obtain a broad overview of a long segment. Software applications have been made available for non-commercial use at http://bioinformatics.ua.pt/software/dna-at-glance.

## Introduction

This paper is about looking at DNA sequences or, more precisely, at graphical representations of DNA sequences. In other words, it is about the summarization of DNA data bearing in mind graphical representations, a problem related to some of the current challenges in large-scale computing [1]. The idea is old, as the sayings “a picture is worth a thousand words” and the century-old advertisement title “one look is worth a thousand words” show. In fact, the association of graphical information to DNA sequences has been pursued for long. Sequence logos [2] and the chaos game representation (CGR) [3] are two well-known examples. Most often, the underlying motivation is to look for and to display information related to the degree of randomness of the sequences, hoping to find meaningful structure. The degree of randomness is intimately related with the complexity, predictability, compressibility, repeatability and, ultimately, with the information theoretic notion of entropy of a sequence. Other methods, use the graphical paradigm for presenting several parameters that can be obtained from a DNA sequence. For example, the Genome Atlas of Jensen *et al.* [4] allows the visualization of information related to repeats, nucleotide composition, and structural parameters, in microbial genomes (the genome of *E. coli* is analyzed in [5] using this approach).

Some methods provide visual information of global properties of the DNA sequences. For example, CGR uses the distribution of points in an image to express the frequency of the oligonucleotides that compose the sequence [6]. From these CGR images, other global representations can be derived, such as genomic signatures [7], [8] or entropic profiles [9].

Originally [9], entropic profiles were estimated using global histograms of the oligonucleotide frequencies, calculated using CGR images. Later, they have been generalized by Vinga *et al.* [10] in order to calculate and visualize local entropic information. Other approaches for estimating the randomness along the sequence have also been proposed. For example, Crochemore *et al.* [11] used the number of different oligonucleotides that are found in a window of predefined size for estimating the entropy. Troyanskaya *et al.* [12] proposed the linguistic complexity, also calculated on a sliding window, as a measure of the local complexity of the DNA sequence.

Both the global and the local estimates of the randomness of a sequence provide useful information and both have shortcomings. The global estimates do not show how the characteristics change along the sequence and the local estimates fail to take into consideration the global properties of the sequence. This last drawback was addressed by Clift *et al.* [13] using the concept of sequence landscape. Using directed acyclic word graphs, they were able to construct plots displaying the number of times that oligonucleotides from the target sequence occur in a given source sequence. If the target and source sequences coincide, then the landscape provides information about self-similarities (repeats) of the target sequence.

The sequence landscapes of Clift *et al.* [13] seem to have been the first attempt of displaying local information while taking into account the global structure of the sequence. This idea was also pursed by Allison *et al.* [14], using a model that considers a sequence as a mixture of regions with little structure and regions that are approximate repeats. Based on this statistical model, they have produced information sequences, which quantify the amount of surprise of having a given base at a given position, knowing the remaining left (or right) part of the sequence. When plotted, these information sequences provide a quick overview of certain properties of the original symbolic sequence, allowing for example to easily identify zones of rich repetitive content [15]–[17].

The interest of complexity measures for DNA sequence analysis has been explored by several researchers, such as in [18]–[20]. The key measure is known as Kolmogorov complexity, and was independently introduced by Solomonoff [21], [22], Kolmogorov [23] and Chaitin [24], and further developed in [25], [26]. The Kolmogorov complexity of a string is the shortest program for a universal computer which outputs the string and stops. This measure is non-computable and is usually approximated by other computable measures, such as, Lempel-Ziv complexity measures [18], [27], linguistic complexity measures [28], or compression-based complexity measures [17], [29], [30].

The information sequences of Allison *et al.* [14] are intimately related to data compression. The importance of data compression for pattern discovery in the context of DNA sequences was already recognized by Grumbach *et al.* [31] and, since then, it has been reinforced by others (e.g. [15], [32]). In fact, the existence of regularities in a sequence renders it algorithmically compressible. The algorithmic information content of a sequence is the size, in bits, of the shortest accurate description of the sequence.

Compression-based complexity measures have an intuitive definition (regular sequences are compressible, as opposed to random ones) and their efficiency is easily quantifiable by the number of bits generated by the encoder. DNA is non-stationary, with regions of low information content (i.e., low entropy or low complexity) alternating with regions of average entropy close to two bits per base. This property is modeled by most DNA compression algorithms with a low-order finite-context model for the high-entropy regions and a Lempel-Ziv dictionary-based or copy-based approach for the repetitive, low-entropy regions. XM [16] has been one of the most successful approaches for compressing DNA sequence data. Also, because it provides a probabilistic distribution for each DNA base being encoded, it offers a natural way of obtaining the above mentioned information sequences [17].

In this paper we “look at DNA sequences” by using information profiles derived from a probabilistic model. The model consists of a combination of several finite-context models, each of a different depth. Such models have been shown to adequately capture the statistical properties of DNA sequences [30], [33], [34] but are direction-dependent, that is, the results depend on which direction the DNA sequence is processed. In this work we remove this directional dependency by combining the amount of information that a certain DNA base carries in each processing direction.

The information profiles are found using an algorithm based on finite-context models that needs time proportional to the length of the sequence. We present a proof-of-concept study of the potential of information profiles in genome analysis, namely, for detecting genomic structural and functional regularities. We uncover genomic regularities on a large-scale, such as, centromeric and telomeric regions of a chromosome, or transposable elements. In this context, we use the genome of the fission yeast *Schizosaccharomyces pombe* strain 972 h^−^ as case-study. We also present the potential of information profiles in a comparative genomics approach, using five human chromosomes 22. This example provides evidence that the proposed method scales well when applied to larger genomic sequences.

## Materials and Methods

### Genomic Data

We use chromosomes I (accession number NC003424.3), II (accession number NC003423.3) and III (accession number NC003421.2) of the genome of the fission yeast *Schizosaccharomyces pombe* strain 972 h^−^ [35], retrieved from the National Center for Biotechnology Information (NCBI) website (http://www.ncbi.nlm.nih.gov/). We also use the human chromosome 22 of the reference genome assembly GRCh37.p9 (accession number NC000022.10, [36]), the alternate Celera assembly (accession number AC000065.1, [37]), the genome of J. Craig Venter (HuRef assembly, accession number AC000154.1, [38]), the genome of a Han Chinese individual (YH assembly, [39]), and the genome of a Korean individual (KOREF 20090224 assembly, [40]). The first three mentioned versions of human chromosome 22 were also retrieved from NCBI, the YH chromosome 22 was retrieved from the Beijing Genomics Institute (BGI) website (ftp://public.genomics.org.cn/BGI/yanhuang/fa/), and the KOREF chromosome 22 was retrieved from ftp://bioftp.org/BiO/Store/Genome/KOREF_KoreanReferenceGenome/KOREF_20090224/fasta/.

### Information Profiles based on Finite-context Models

To “look at DNA” at different scales we rely on information profiles that quantitatively measure the local complexity of the DNA sequence. The profiles provide a visual representation of the sequence, and can be interpreted in a simple way. The less regular the behaviour, the higher the numerical values. Thus, visual inspection immediately shows regions of low complexity (for example, repetitions), regions of high complexity, and other patterns of possible interest.

The probabilistic models required to draw the information profiles are, not surprisingly, related to data compression and information-theoretic concepts such as entropy. Compression and modeling are intertwined: the problem of discovering an efficient representation of the information source can be stated as a data modeling problem.

A probabilistic model of a DNA sequence is a mathematical description of the sequence, viewed as an information source. The model provides an estimate of the probability of the next DNA symbol. The entropy of the model sets a lower bound on the compression performance. Conversely, compression performance yields a bound on the entropy. However, a given compression method may have limited potential or interest in connection with information profiles.

Of the myriad of coding methods proposed for compressing genomic sequences (e.g. [16], [30], [31], [33], [34], [41]–[49]), most are based on search procedures for finding exact or approximate repeats, in the sequence itself or in its reversed complement. Although this may lead to interesting compression rates, it generally requires a significant computational effort. Recently, it has been shown that appropriate combinations of finite-context models are able to give competitive [30] or even superior [34] compression results, at a smaller computational cost.

Finite-context models are probabilistic models based on the assumption that the information source is Markovian, i.e., that the probability of the next outcome depends only on some finite number of (recent) past outcomes referred to as the context. The proposed approach is based on a mixture of finite-context models. We assign probability estimates to each symbol in 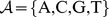, regarding the next outcome, according to a conditioning context computed over a finite and fixed number *k*>0 of past outcomes 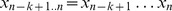 (order-*k* finite-context model with 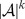 states).

The probability estimates 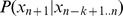 are calculated using symbol counts that are accumulated while the sequence is processed, making them dependent not only on the past *k* symbols, but also on *n*. We use the estimator
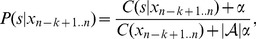
(1)where 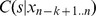 represents the number of times that, in the past, symbol *s* was found having 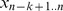 as the conditioning context and where
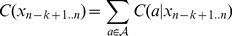
(2)is the total number of events that has occurred so far in association with context 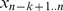. Parameter α allows balancing between the maximum likelihood estimator and an uniform distribution (when the total number of events, n, is large, it behaves as a maximum likelihood estimator). For α = 1, (1) reduces to the well-known Laplace estimator.

The per symbol information content average provided by the finite-context model of order-*k*, after having processed *n* symbols, is given by
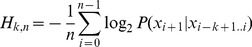
(3)bits per symbol. When using several models simultaneously, the 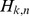 can be viewed as measures of the performance of those models until that instant. Therefore, the probability estimate can be given by a weighted average of the probabilities provided by each model, according to
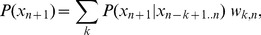
(4)where 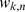 denotes the weight assigned to model k and



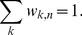
(5)Our modeling approach is based on a mixture of probability estimates. In order to compute the probability estimate for a certain symbol, it is necessary to combine the probability estimates given by (1) using (4). The weight assigned to model *k* can be computed according to
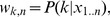
(6)i.e., by considering the probability that model *k* has generated the sequence until that point. In that case, we would get
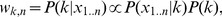
(7)where 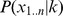 denotes the likelihood of sequence 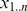 being generated by model k and 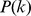 denotes the prior probability of model k. Assuming
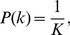
(8)where K denotes the number of models, we also obtain



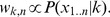
(9)Calculating the logarithm we get
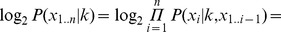
(10a)

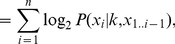
(10b)which is related to the number of bits that would be required by model *k* for representing the sequence 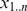. It is, therefore, the accumulated measure of the performance of model *k* until instant *n*. DNA sequences are known to be non-stationary. Due to this, the performance of a model may vary considerably from region to region of the sequence. In order to extract the best possible performance from each model, we adopted a progressive forgetting mechanism. The idea is to allow each model to progressively forget the distant past and, consequently, to give more importance to recent outcomes. Therefore, we rewrite (11) as



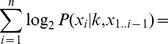
(11a)

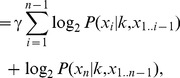
(11b)where 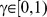 dictates the forgetting factor to be used. Defining
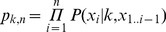
(12)and removing the logarithms, we can rewrite (0) as
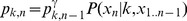
(13)and, finally, set the weights to



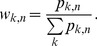
(14)This probabilistic model yields an estimate of the probability of each symbol in the DNA sequence, and as such it allows us to quantify the degree of randomness or surprise along one direction of the sequence.

## Results and Discussion

Chromosomes are processed both in the downstream, or direct (5′→3′), and upstream, or reversed (3′→5′), directions. This dual processing aims at eliminating the directionality bias introduced when only one of the two possible directions is taken into consideration. Therefore, the information content of each DNA base is calculated by running the statistical model in one direction, then in the other direction, and finally by taking the smallest value obtained.

### Fission Yeast

**Figure 1:**
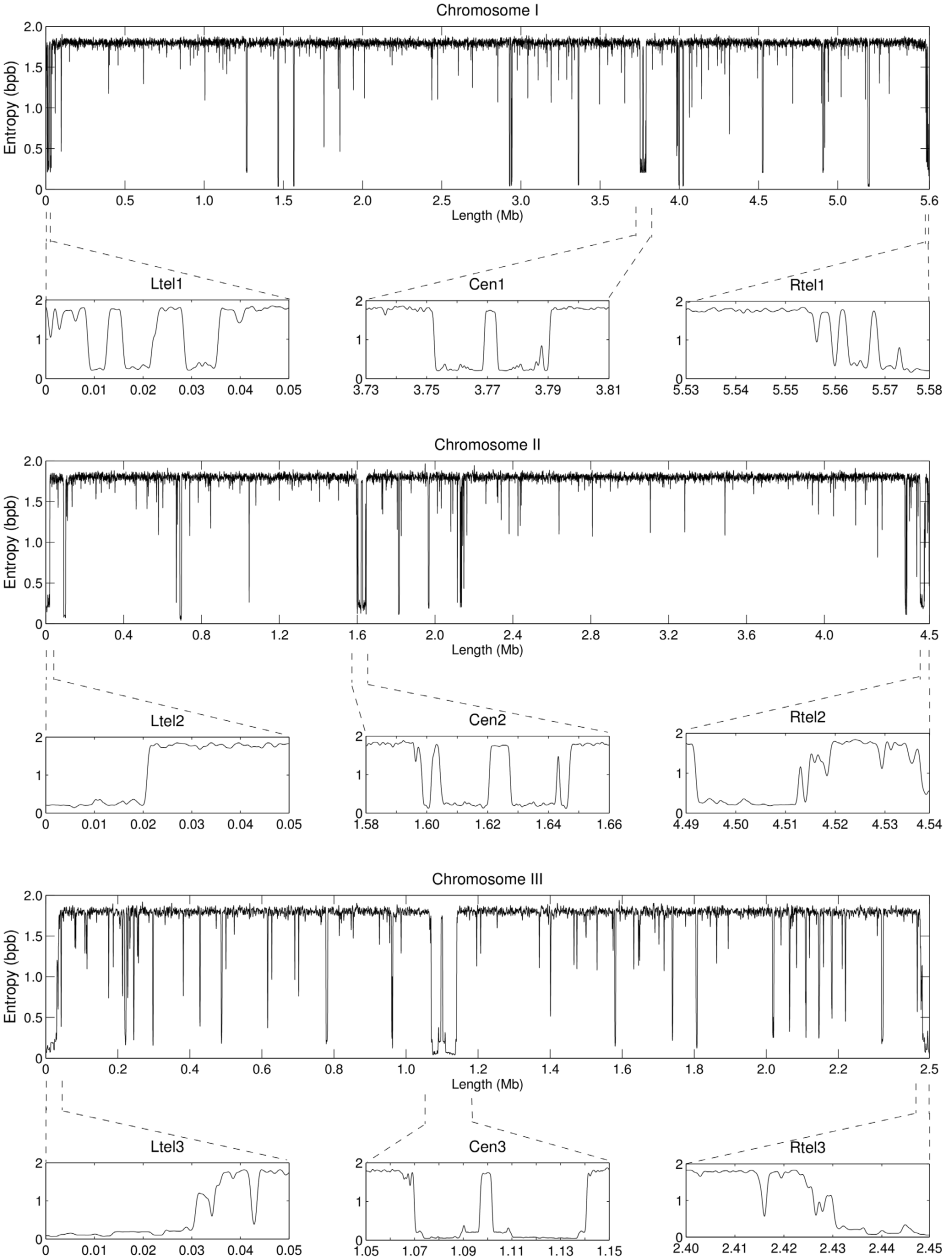
Information profiles of the chromosomes of *S. pombe* highlighting centromeric and telomeric regions. The profiles are the result of eight competitive finite-context models with context depths 2, 4, 6, 8, 10, 12, 14 and 16. They represent the minimum of the combined direct and reversed profiles, low-pass filtered with a Blackman smoothing window of 1,001 bp. Probabilities were estimated with 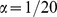 for contexts 14 and 16. For clarity, the full chromosome profiles were sampled every 20 bp. Zoomed in profiles Ltel and Rtel display telomeric and subtelomeric regions of each chromosome, and zoomed in Cen profiles display the respective centromeric regions.

Figure 1 displays the information profiles of the three chromosomes in the genome of *S. pombe*, obtained independently for each chromosome. The profiles are the result of the combination of eight finite-context models with context depths of 2, 4, 6, 8, 10, 12, 14 and 16. They represent the minimum of the combined direct and reversed profiles, low-pass filtered with a Blackman window of 1,001 bp. Probabilities were estimated with 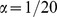 in Eq. 1 for the larger contexts of *k* = 14 and *k* = 16. For clarity, the full chromosome profiles shown result from sampling every 20 bp.

Low-information regions in Fig. 1 are associated with the presence of repetitive sequences. For example, chromosome III has more and often more prominent low-information regions than chromosomes I and II, which is in compliance with some properties of this chromosome concerning repetitive structures, such as, the presence of tandem rDNA repeats [50] or the density of transposable element remnants in this chromosome being twice that of chromosomes I and II [35]. Annotated with Ltel and Rtel are regions of low-information content pertaining the telomeric (where available; see http://www.sanger.ac.uk/Projects/S_pombe/telomeres.shtml) and subtelomeric regions of each chromosome. Annotated with Cen are regions of low-information content pertaining the centromeric regions of each chromosome.

Telomeres in *S. pombe* consist of ∼300 bp long tandemly repeated 5′-GGTTACA_0−6_C_0−1_G_0−6_-3′sequences, with the GGTTAC repetitive unit being the most commonly found [51], [52]. Chromosomes I and II share some subtelomeric sequences, while the telomeric repeats at both ends of chromosome III are immediately flanked by tandem arrays of rRNA genes [50], [53]. The highly repetitive content of these regions, including degenerate and tandem telomeric repeats [50], and duplicated and highly-similar subtelomeric regions [35], [50], is captured in the low-information Ltel and Rtel regions of the profiles in Fig. 1.

Mammalian centromeres contain a large number of tandemly arranged repetitive sequences. Wood *et al.* [35] reported an estimated length of 35 kb for the centromere of chromosome I, 65 kb for the centromere of chromosome II, and 110 kb for the centromere of chromosome III, in inverse proportion to the lengths of the respective chromosomes, namely, 5.7 Mbp, 4.6 Mbp, and 3.5 Mbp. However, updated centromere positions are cen1: 3,753,687–3,789,421 bp, cen2: 1,602,264–1,644,747 bp, and cen3: 1,070,904–1,137,003 bp (http://www.sanger.ac.uk/Projects/S_pombe/centromere.shtml), which correspond to a decrease of ∼30% in the length of annotated centromeric regions cen2 and cen3 with respect to previous values [35]. These updated lengths of the centromeric regions and inverse proportionality to the chromosome size are recovered in the information profiles of Fig. 1. Cen1 consists of a non-conserved central core (cnt1) of 4.1 kb flanked by two 5.6-kb imperfect inverted imr1 repeats (imr1L, imr1R) that display sequence identity with each other, and two pairs of 4.4-kb dg and 4.8-kb dh repeats (dg1, dh1) separated by cen253, a repeat of ∼0.3 kb. The maps of the other two centromeres have the same basic structure, with central cnt regions flanked by imr repeats and by variable numbers of dg and dh repeats separated by cen253. Moreover, there are many tRNA genes in the centromeric regions, with clusters flanking cen2 and cen3 and also within the imr regions of all three centromeres [35], [52]. This centromeric mirror-like repetitive structures are captured in the Cen regions of the profiles in Fig. 1, with the core cnt regions evident by higher-information central peaks, the imr and dg/dh repeats accounting for regions of low-information content, and t-RNA genes contributing to other peaks of higher-information e.g. at the frontiers of cen2.

**Figure 2:**
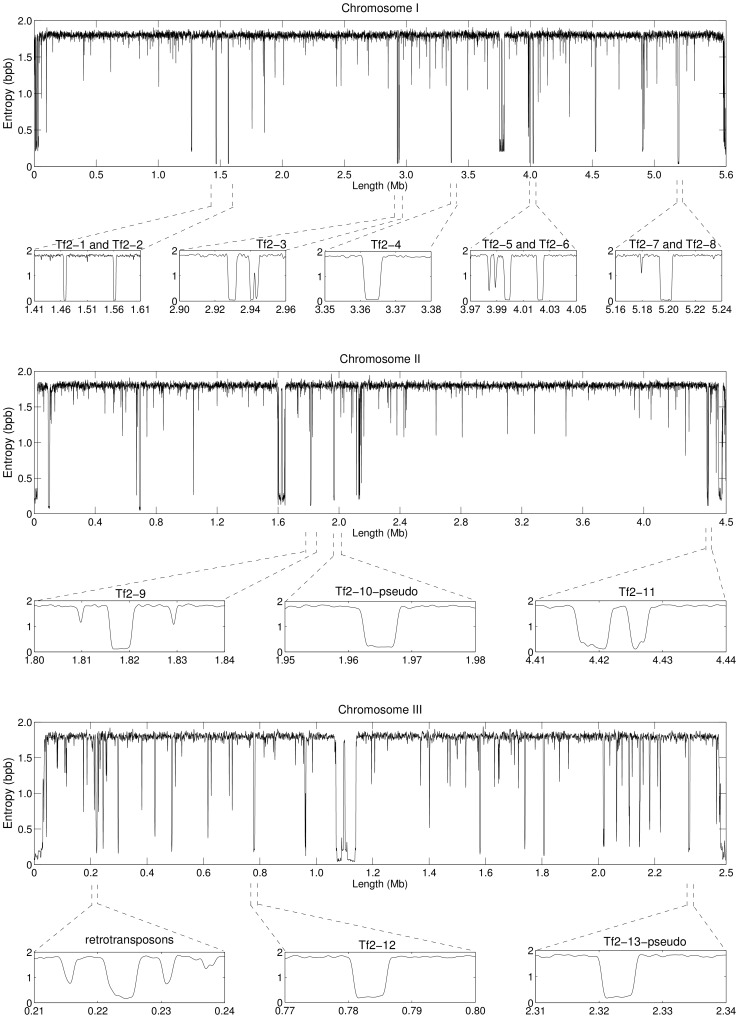
Information profiles of the chromosomes of *S. pombe* highlighting Tf2-type retrotransposons. Parameters are the same as in Fig. 1. Zoomed in profiles display the 13 full length Tf2 elements and an additional display of other Tf2-type retrotransposons.

Figure 2 displays again the information profiles of the three chromosomes in the genome of *S. pombe*, obtained and sampled similarly as in Fig. 1. Highlighted are again low-information regions associated with the presence of repetitive sequences, now focusing on transposable elements.

Two related families of long terminal repeat (LTR)-retrotransposons, named transposon of fission yeast 1 (Tf1) and 2 (Tf2), have been identified in *S. pombe* [54]. Retrotransposons are mobile DNA elements ubiquitous in eukaryotic genomes, which remain active in most mammalian genomes. They mobilize via an RNA intermediate that is then reverse transcribed and reintegrated into the genome by a copy-and-paste mechanism, thereby duplicating the element. Strain 972 h^−^ of *S. pombe* contains 13 full length Tf2 elements of length ∼4.9 kb and no Tf1 elements. It also contains many single LTRs derived from Tf1 and Tf2 elements [52]. Annotated in Fig. 2 are all 13 full length Tf2 elements, plus an additional display of other Tf2-type retrotransposons. Plot Tf23 in chromosome I displays that retrotransposon in the first low-information region, followed by a large retrotransposon in the second low-information region, which is in accordance with the annotations in [52]. The wider low-information region in plot Tf27 and Tf28 includes both elements. Plot Tf211 in chromosome II displays that retrotransposon in the second low-information region, preceded by a large retrotransposon. The plot annotated with retrotransposons in chromosome III showcases some of the LTRs derived from Tf1 and Tf2 elements, where a large repeat is associated to the wider low-information region and two smaller elements are identified in the two additional low-information regions.

This accurate matching of the low-information regions in Figs. 1 and 2 to annotated repetitive genomic structures, such as the centromeric and telomeric regions of a chromosome or its transposable elements, proves information profiles may be useful in *de novo* discovery of large-scale genomic regularities. Clearly, it is not possible to infer the genomic sequence *per se* from the information profiles, or the location of genomic regularities within base pair resolution. However, it is possible to discover the presence of regularities on a genome-wide scale, which may be useful for an exploratory genome analysis or for genome comparisons.

### Human Chromosome 22

To illustrate the potential of information profiles in the analysis of larger and more complex genomes, we use the human chromosome 22 as case-study. This ∼51 Mbp chromosome is the second smallest human autosome and it was the first to be fully sequenced [55].

**Figure 3:**
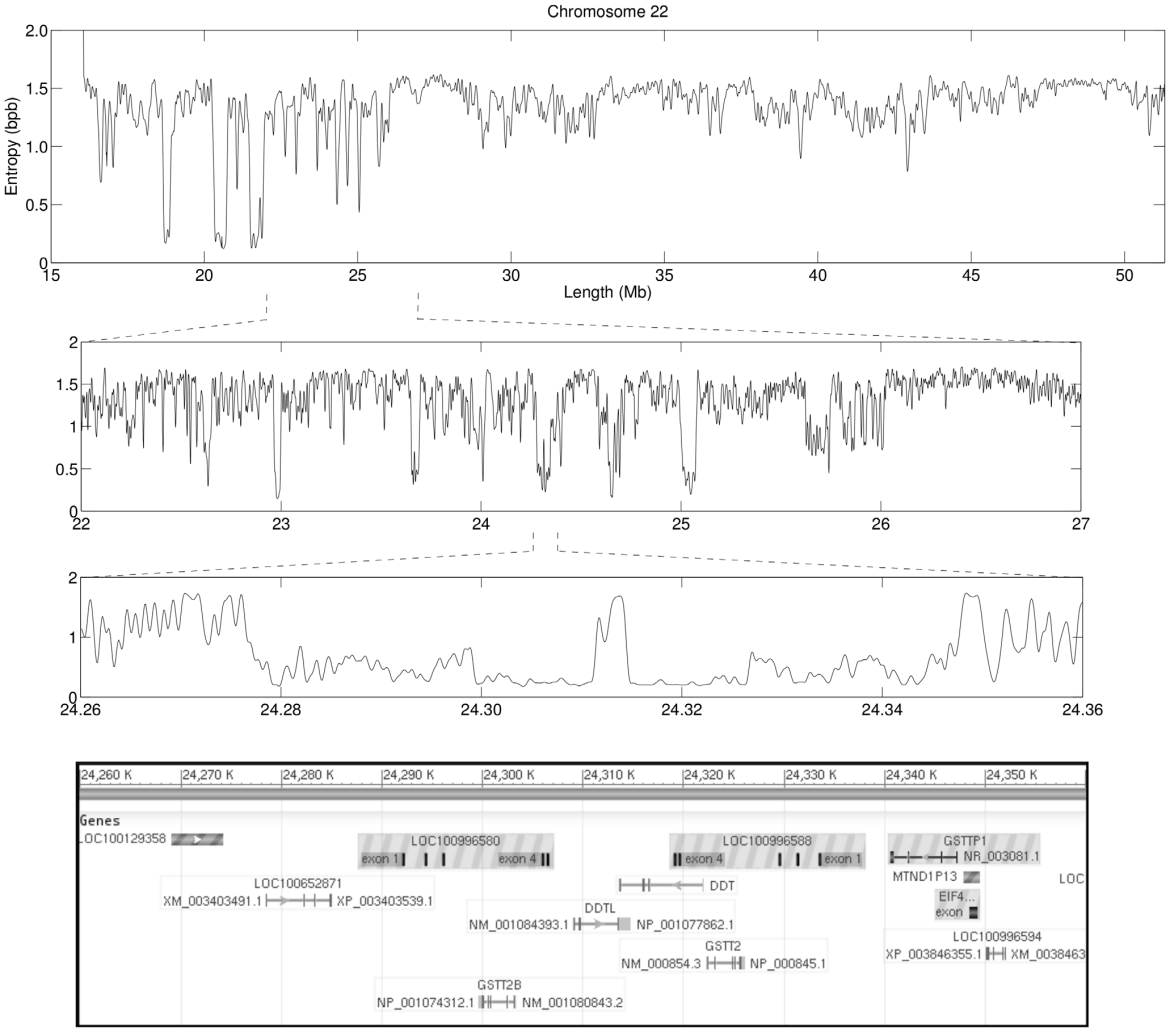
Information profile of chromosome 22 of the GRCh37 human reference genome assembly. Parameters are similar to those of Fig. 1, except for the smoothing window, which has a value of 100,001-in profiles reveal regularities at increasingly larger resolution, including several genes from duplicated gene families in the lower plot. The lower panel was downloaded from the NCBI website and it identifies annotated genes in the zoomed-in region.

Figure 3 displays the information profile of chromosome 22 of the GRCh37 reference human genome assembly. As before, the profiles are the result of the combination of eight finite-context models with context depths of 2, 4, 6, 8, 10, 12, 14 and 16. They represent the minimum of the combined direct and reversed profiles, low-pass filtered. Probabilities were estimated with 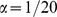 in Eq. 1 for the larger contexts of *k = *14 and *k = *16. As the first 15 Mbp remain unsequenced (containing solely Ns), the upper plot in Fig. 3, which displays the information profile of the whole chromosome, ignores this region. In order to display the global information of such a large chromosome, the profile should be heavily low-pass filtered. Here, we used a smoothing window size of 100,001 bp.

The first striking feature of this chromosome-wide profile is that the average information content is considerable lower than that of the chromosomes of *S. pombe* (Figs. 1 and 2). This is a direct consequence of the fact that ∼42% of the human chromosome 22 comprises interspersed and tandem repeats [55].

The middle plot in Fig. 3 shows a 5 Mbp zoomed-in region of the chromosome (22–27 Mbp), filtered with a smoothing window of 10,001 bp. Clearly, additional detail and regularities are observable at increasingly larger resolution. Highlighted in the lower plot is a region of low entropy, as consequence of being densely occupied by genes from duplicated gene families, filtered with a smoothing window of 1,001 bp. For completeness, the final plot in Fig. 3 shows an image of gene annotations taken from the NCBI nucleotide browser corresponding to the displayed region. One example of such gene families are the glutathione S-transferases, with several genes and pseudogenes annotated to this region. Gene LOC100652871, a glutathione S-transferase theta-4-like, is located in region 24,278,480–24,284,985 bp. Another glutathione S-transferase theta-4-like gene, LOC100996594, is located in region 24,350,125–24,352,036 bp of the reversed complement. Gene LOC100996580, a glutathione S-transferase theta-1-like, is located in region 24,292,105–24,306,644 bp. Another glutathione S-transferase theta-1-like gene, LOC100996588, is located in region 24,319,105–24,333,620 bp of the reversed complement. Gene GSTT2 (ID 2953), a glutathione S-transferase theta 2, is located in region 24,322,314–24,326,106 bp. Gene/pseudogene GSTT2B (ID 653689), a glutathione S-transferase theta 2B is located in region 24,299,601–24,303,368 bp of the reversed complement. Finally, the pseudogene GSTTP1 (ID 25774), a glutathione S-transferase theta pseudogene 1, is located in region 24,340,595–24,347,258 bp of the reversed complement.

We selected this particular region of chromosome 22 for showcasing because it reinforces the reason why this new tool may be valuable. During our own exploration of this analysis method, we were browsing through the information profile of the human chromosome 22, observing regions of roughly 1 Mbp in length, when that striking and almost symmetric profile of about 100,000 bp caught our attention. Many more similarly interesting regions can be observed along the chromosome. Hence, the tool here proposed provides a handy procedure for quickly detecting potentially interesting genomic regions.

To illustrate the potential of information profiles in the context of comparative genomics, we use again the human chromosome 22 as case-study.

**Figure 4:**
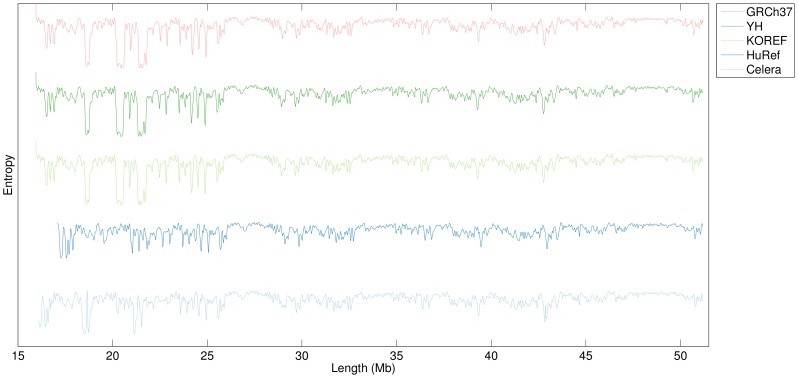
Information profiles of chromosome 22 in five human genome assemblies. Parameters are similar to those of Fig. 1, except for the smoothing window, which has a value of 100,001(e.g., in the range 20–23 Mb).

Figure 4 displays the information profiles for five human chromosomes 22. As before, the profiles are the result of the combination of eight finite-context models with context depths of 2, 4, 6, 8, 10, 12, 14 and 16. They represent the minimum of the combined direct and reversed profiles, and low-pass filtered with a smoothing window of 100,001 bp. Here, similarities and differences between the sequences are clearly visible. Both YH and KOREF human genome assemblies were obtained from resequencing experiments that used the GRCh37 reference human genome assembly for mapping their short reads. This is the main reason why both profiles are so similar to that of GRCh37. On the other hand, the HuRef and Celera assemblies are two *de novo* assemblies [37], [38], hence their profiles are considerably more dissimilar to that of GRCh37.

**Figure 5:**
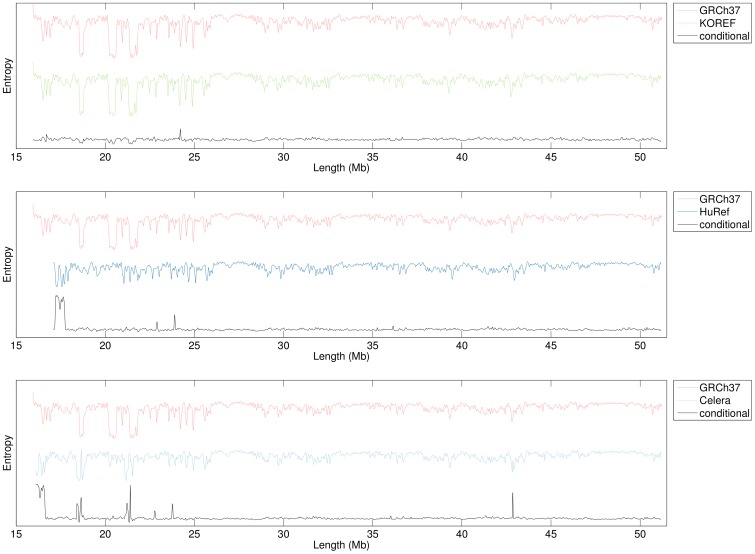
Information and conditional profiles of three pairwise comparisons of human chromosomes 22. Parameters are similar to those of Fig. 1, except for the smoothing window, which has a value of 100,001-context models trained over the GRCh37 human genome assembly. Peaks in these profiles highlight regions of sequence divergence in the KOREF, HuRef and Celera chromosomes, with respect to the GRCh37 one. As an example of the additional information conveyed by these conditional profiles, we highlight the peak in the Celera assembly around base 43 Mb. Whereas slightly perceivable in the non-conditional profiles, the divergence of the two assemblies (GRCh37 and Celera) at this particular location is much more evident in the conditional profile.

Figure 5 repeats the information profiles for four human chromosomes 22, as well as, the conditional profiles of three pairwise comparisons between those chromosomes. Here, the discovery strategy was different, as the conditional profiles were obtained using the statistics of the finite-context models trained over the GRCh37 chromosome, with the same parameters as described above. As such, the baseline-like regions highlight sequence similarity between both chromosomes, whereas peaks highlight regions of clear sequence divergence in the KOREF, HuRef and Celera chromosomes, with respect to the GRCh37 one. The main observation stemming from these conditional profiles pertains the large-scale structural variation between these human chromosomes. For the *de novo* assemblies (HuRef and Celera), most of the observed variation occurs in the beginning of the profiles. The peak a little over the 24 Mb mark is common to all three conditional profiles, hinting at the possibility of this being a highly-variable region in the human population.

## Conclusion

We introduced an algorithm to detect genomic regularities within a *blind* discovery strategy. The algorithm uses information profiles built using an efficient DNA sequence compression method. The results described support our claim that information profiles provide a valuable discovery tool for the genome-wide individual or comparative analysis of genomes, through the detection of biologically-relevant genomic regularities. We used the genome of the fission yeast *Schizosaccharomyces pombe* strain 972 h^−^ for illustration. This model-organism was chosen because of its genome size, which renders visualization of the information profiles easier. Nevertheless, to give evidence that the tool is also applicable to larger genomes, we included information profiles at several scales of the human chromosome 22. Using five human chromosomes 22, we also showcased the potential of this methodology for comparative genomics analyses.

Our algorithm relies on the efficient probabilistic modeling of the genomic sequence based on finite-context (Markov) models. The approach is sufficiently flexible and powerful to enable addressing various biological questions and quickly obtaining the corresponding information profiles for a first-hand assessment. Indeed, the creation of information profiles does not require unusual computational facilities. Building an information profile requires a computation time that varies only linearly with the size of the sequence. For example, the information profile of human chromosome 22 was created in a laptop computer in less than five minutes. Moreover, the amount of computer memory required does not depend on the size of the sequence, but only on the depth of the finite context models used for modeling the sequence.

To facilitate the exploration of the information profiles here introduced, we made available two software applications: one is highly flexible but command-line based; the other has a graphical user interface and was designed to be very easy to use. Both applications are freely available for non-commercial use and can be downloaded from http://bioinformatics.ua.pt/software/dna-at-glance.

Due to its exploratory nature, these software applications currently offer a number of options that allow for many combinations of the parameters. Nevertheless, for ease of use, they can also be ran with default parameters. A detailed explanation of these parameters and some examples of their use is included in the packages.

**Figure 6:**
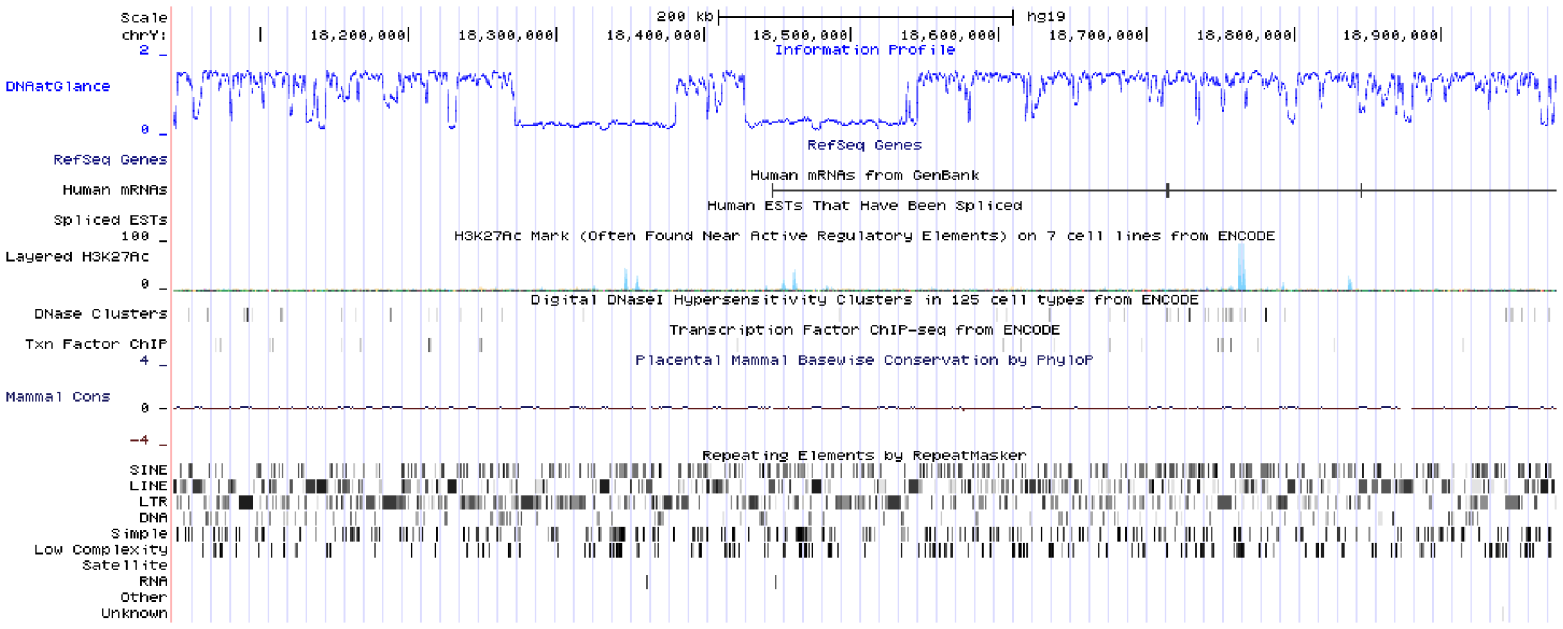
Information profile of part of human chromosome Y integrated in the UCSC Genome Browser. The custom track named “DNAatGlace” was uploaded to the browser in WIG format.

In Figure 6 we show an example of integration of the information profiles in the UCSC Genome Browser, in this case displaying a segment of human chromosome Y. The data were uploaded as a custom track in WIG format (that can be produced by the supporting software applications). As can be seen in this example, there are two relatively large regions of low information content that are not easily guessed by inspection of the output provided by the RepeatMasker tool, giving evidence of the complementary nature of the approach described in this paper.

The ability “to look at a DNA sequence” and immediately being able to visually identify regions of potential interest is, in our opinion, a valuable tool for the biologist. The work that we present in this paper is an important step in that direction.
